# Drivers of Viral Spillover: An Examination of How Pathogens Spread

**DOI:** 10.3390/pathogens15090901

**Published:** 2026-08-26

**Authors:** Elenoire Sole, Giulia Montalbano, Giuseppe Motta, Maria Maddalena Pansera, Angelina Midiri, Mariarita Iacopino, Paolo Liotta, Giuseppe Mancuso, Carmelo Biondo

**Affiliations:** Department of Human Pathology, University of Messina, 98125 Messina, Italy; elenoiresole@icloud.com (E.S.); giuliamontalbano6@gmail.com (G.M.); giuseppemotta94@gmail.com (G.M.); panseramarilena@gmail.com (M.M.P.); amidiri@unime.it (A.M.); mariarita.iacopino@studenti.unime.it (M.I.); paolo.liotta@studenti.unime.it (P.L.); cbiondo@unime.it (C.B.)

**Keywords:** viral adaptation, zoonotic diseases, climate change, one health framework

## Abstract

The occurrence of viral zoonotic spillover (the transmission of viruses from animals to humans) has attracted worldwide attention due to mounting concerns regarding viral threats such as avian influenza, Hendra, monkeypox, Nipah and bat coronaviruses. It is evident that these events deviate significantly from natural occurrences, being the conflation of ecological, environmental and social factors that are profoundly altering the boundaries between the animal and human domains. Consequently, in order to comprehend both the contemporary and prospective drivers of zoonotic viral spillover, a coordinated, comprehensive global One Health response is essential. The purpose of the present review is to analyze prevailing pathways of transmission between animal reservoirs and human populations. This examination involves the analysis of historical cases, including those of SARS and Ebola, as well as recent occurrences, such as the global pandemic of SARS-CoV-2 (COVID-19). A secondary objective of this review is to comprehend the fundamental mechanisms of cross-species transmission. The development of effective strategies to mitigate the emergence of zoonotic viruses and prevent future pandemics is contingent on a robust understanding of these mechanisms. This analysis is key to identifying pandemic pathogens and preventing the spread of zoonotic viruses. In conclusion, the review provides a thorough evaluation of current global strategies to prevent the spread of zoonotic viruses, highlighting gaps in our understanding and areas for further research.

## 1. Introduction

**Figure 1:**
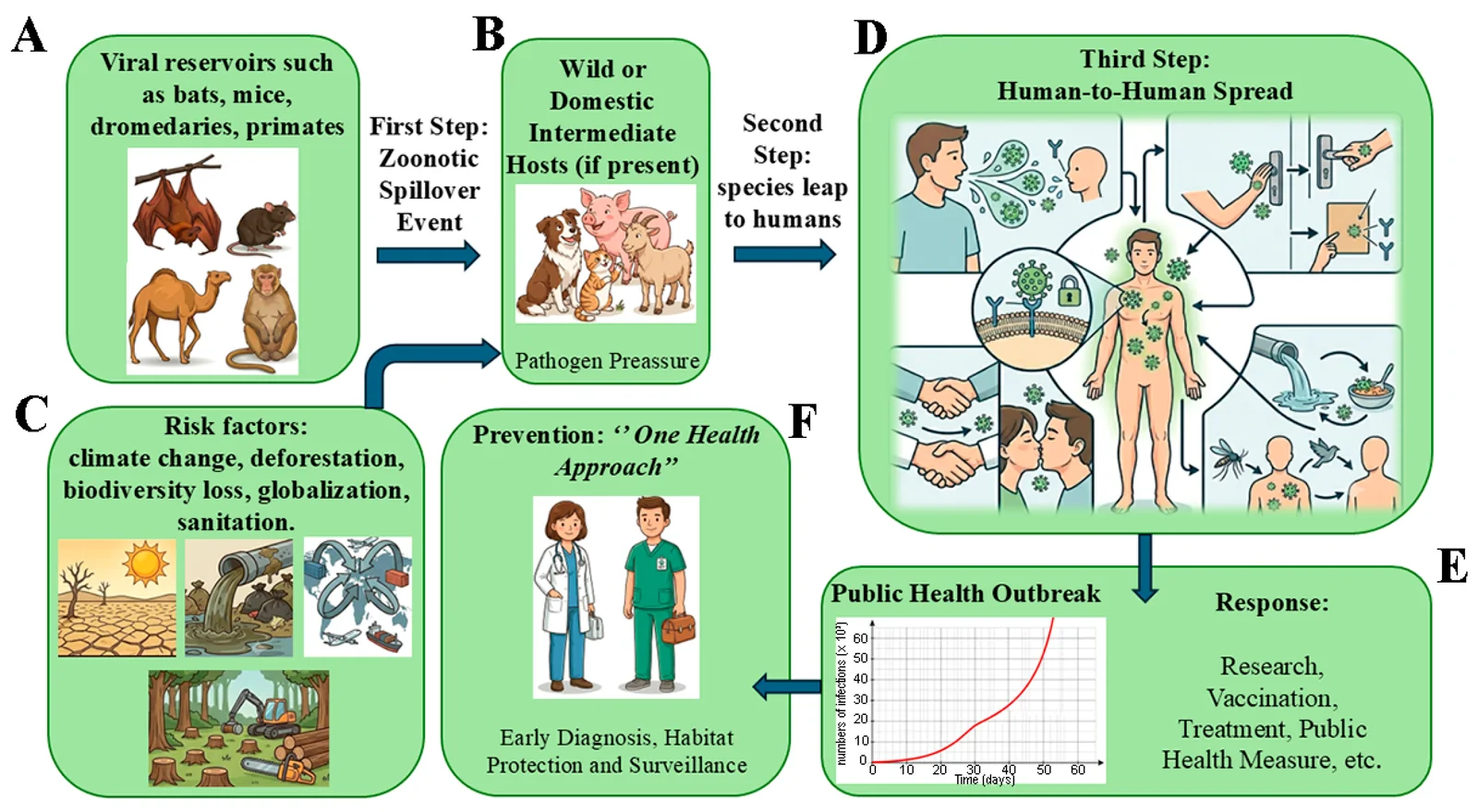
Schematic representation of the viral spillover process. The diagram illustrates the sequential stages of a zoonotic event, detailing the transmission of pathogens from natural reservoirs (such as primates, bats, rodents, and dromedaries) (**A**) to intermediate hosts (**B**), where applicable [8]. Anthropogenic and environmental factors, such as globalization, climate change, and biodiversity loss, exert selective pressures that facilitate this cross-species jump [7] (**C**). The virus ultimately transmits from animals to humans, with subsequent human-to-human transmission potentially culminating in an epidemic [9] (**D**). Mitigation and prevention strategies are based on targeted research, therapeutics, early diagnosis, and vaccination [10] (**E**). Furthermore, public health frameworks such as the “One Health” approach play a pivotal role in strengthening defense mechanisms by facilitating close collaboration among healthcare professionals and epidemiological surveillance networks [11] (**F**).

The World Health Organization (WHO) defines a ‘zoonosis’ as an infectious disease of animal origin with the capacity to be transmitted to humans, often via direct contact, contaminated food or the environment, caused by one of four main pathogenic agents: viruses (e.g., *Ebola*, *Rabies*), bacteria (e.g., *Bacillus anthracis*, *Salmonella*), parasites (e.g., *Plasmodium*) or fungi (e.g., *Microsporum)* [1]. Furthermore, approximately 60% of known infectious diseases and up to 75% of new ones have a zoonotic origin. Annually, approximately one billion cases of illness and more than 2.5 million of deaths worldwide are attributable to zoonoses [2]. Zoonotic transmission is a phenomenon that has been observed throughout history, as evidenced by historical pandemics which demonstrate that scourges, such as those caused by *Yersinia pestis,* have afflicted humanity for millennia [3]. The history of plague is marked by three major outbreaks. The Plague of Justinian (541–750 AD) is widely regarded as the first documented pandemic of bubonic plague, resulting in the deaths of 25–60% of the European population at the time. The second pandemic became known as the Black Death, killing an estimated one-third of Europeans between 1346 and 1352. The third pandemic began in 1772 in Yunnan and spread globally in the 20th century [4]. However, recent scientific findings suggest that plague lineages were already in circulation and causing zoonotic outbreaks as early as 5000 years ago, at the time of the Neolithic period, when animal husbandry was being adopted [5]. While a wide array of pathogens has been identified as causative agents for the emergence of zoonotic diseases, including bacteria (e.g., *Salmonella*), parasites (e.g., *Echinococcus* spp.), and fungi (e.g., *Cryptococcus* spp.), viruses have been recognized as the most prevalent emerging zoonotic agents responsible for the majority of recent global epidemics (e.g., SARS-CoV-2, Ebola, Nipah, MERS) [6]. A multitude of human activities, including deforestation, biodiversity loss, wildlife trade, intensive farming and urban expansion, are causing ecosystems to break down, thereby increasing the risk of spillover [7] (Figure 1).

The term ‘zoonotic spillover’ refers to the transmission of pathogens from animals to humans. It has been estimated that this process accounts for approximately 60% of all recognized infectious diseases, and as much as 75% of new or emerging infectious diseases in human populations [2,8]. While the efficacy of preventative and surveillance measures in detecting zoonotic viral outbreaks is fundamental, these measures are frequently characterized by a lack of coordination and resources at the human–animal–environment interface. The objective of the present review is to understand how ecological, biological and socio-economic factors contribute to spillover events and pandemic risks. It also examines the transmission of diseases between species, drawing upon outbreaks such as Ebola and SARS to illustrate the current strategies employed, the research required and the value of One Health approaches. A search was conducted on Medline, Scopus and Web of Science using the search terms “spillover”, “zoonotic transfer”, “zoonotic spillover”, “pathogen spillover”, and “cross-species transmission”. We also considered articles published in English, regardless of year of publication, focusing on animal–human interface prevention and zoonoses.

## 2. Ecological Drivers and Predictive Modeling of Zoonotic Spillover

Deforestation, habitat fragmentation, biodiversity loss and climate change act as anthropogenic accelerators of zoonotic viral spillover risk [12,13]. The degradation of primary ecosystems has the effect of generating extensive ecological edge habitats, with the result that contact frequency and proximity among wildlife, domestic animals and human populations are significantly increased [13,14]. Furthermore, the deprivation of nutrients resulting from the destruction of habitats leads to the induction of physiological and immunological stress in species inhabiting reservoirs, thereby triggering endogenous viral replication and increasing the rate of pathogen shedding into the environment [14,15]. In intact, biodiverse contexts, the presence of a variety of low-competence host species exerts an epidemiological containment, limiting pathogen circulation. Ecosystem simplification selects for generalist and opportunistic species (e.g., rodent families) with high reservoir competence, increasing infection prevalence and the likelihood of spillover to humans [12,16]. Rising global temperatures and altered precipitation regimes are reshaping ecological niches, driving species to higher or lower latitudes. This is facilitating new interspecific interactions and the spatiotemporal expansion of arthropod vectors [17]. Using environmental data in advanced predictive models has greatly improved how quickly outbreaks can be identified. For example, recent analyses of the Ebola outbreak in Africa combined data on forest loss, population density and weather to estimate risk [18]. Empirical evidence shows a strong link between local habitat fragmentation and the likelihood of spillover. Using these tools, we can identify high-risk areas and develop targeted prevention strategies within a One Health approach [18].

## 3. Major Zoonotic Viral Outbreaks in the Twenty-First Century Are Spillovers from Animals to Humans

**Figure 2:**
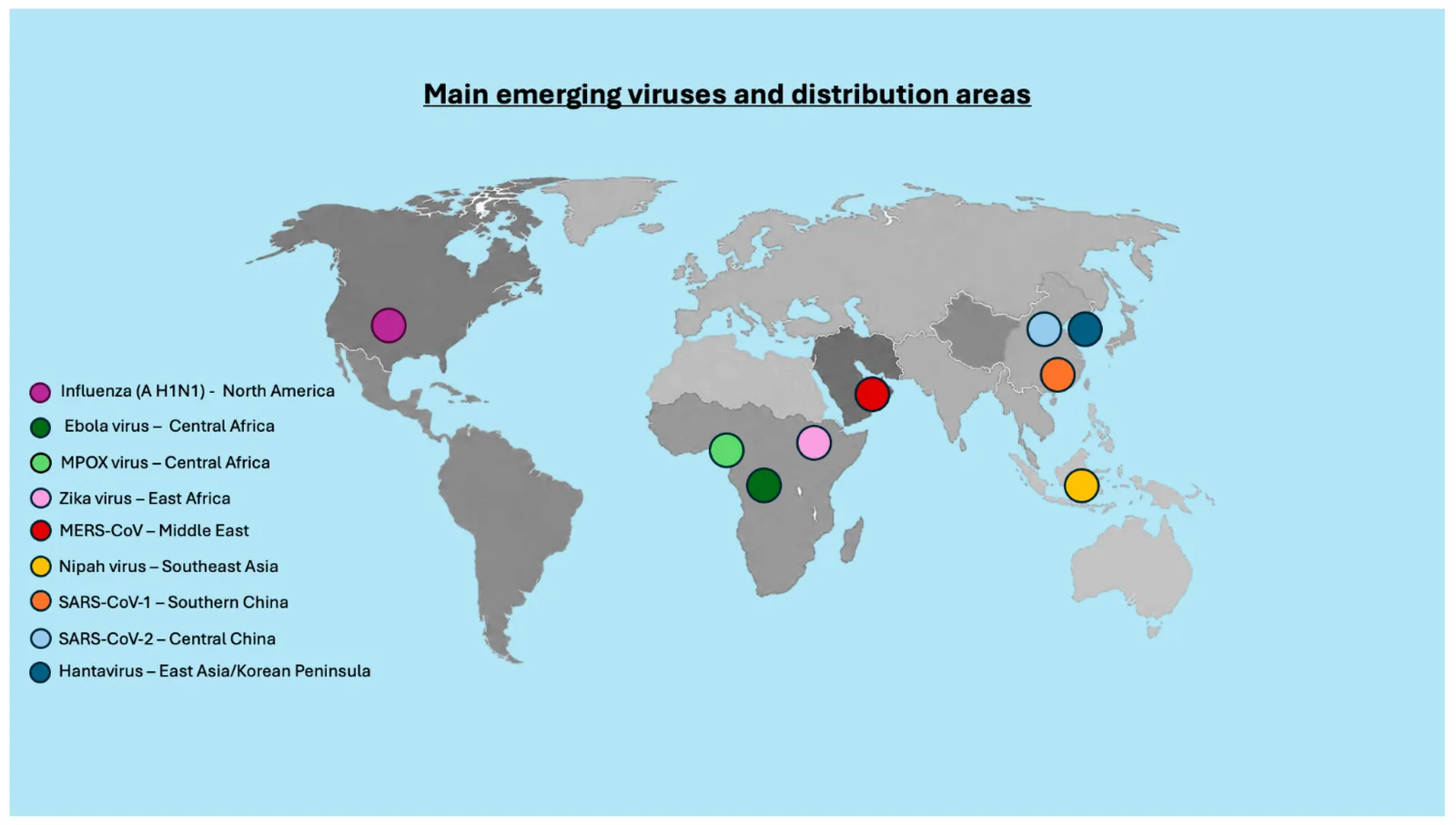
Global mapping of major emerging viral threats (highlighting locations of first reported cases). Geographic localization of key viral emergence zones: influenza A (H1N1) in North America [32]; Ebola and MPOX viruses in Western and Central Africa [33,34]; Zika virus in East Africa [23]; MERS-CoV in the Middle East [35]; Nipah virus in Southeast Asia [36]; SARS-CoV-1 and SARS-CoV-2 in Southern and Central China [37]; and hantavirus in East Asia and the Korean Peninsula [38]. The highlighted areas show where the first cases of the emerging viruses were reported.

The WHO constantly monitors viruses of zoonotic origin that threaten global health [19]. They cause 2.5 billion cases of illness and 2.7 million deaths a year. This is nearly 60% of all infectious diseases and 75% of new infections [2,8]. The most significant zoonotic viral threats identified by the WHO are outlined in Table 1 and Figure 2.

**Figure 3:**
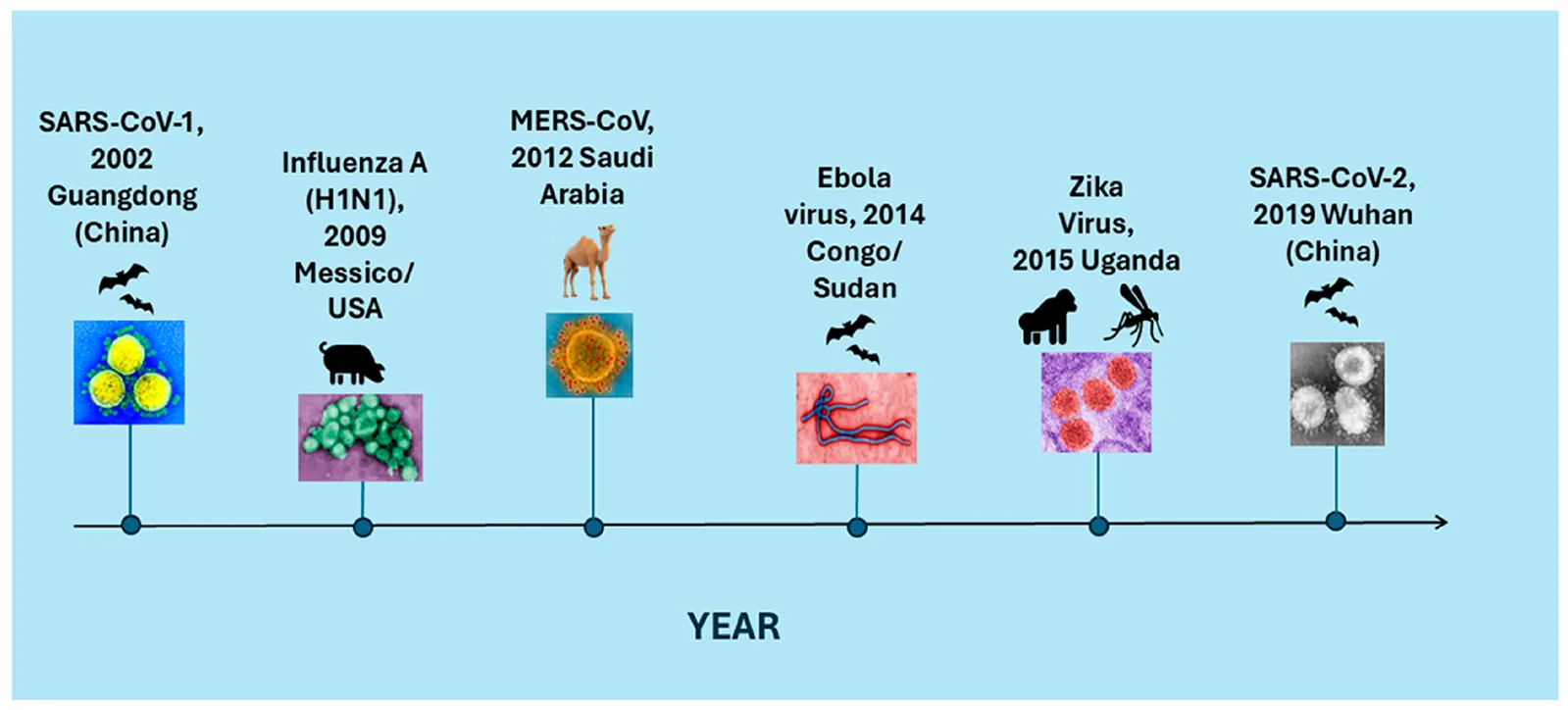
Discovery of emerging viruses in the 21st century.

As shown in Table 1, zoonoses may present in two distinct forms: direct, whereby the pathogen is transmitted directly from animals to humans, or indirect, which necessitates the involvement of a vector in its transmission [1]. The latter, also known as vector-borne diseases (VBDs) have attracted increasing attention due to their spread, as evidenced by the outbreaks of Chikungunya and Zika virus in the American region in 2014 and 2015 [39]. Furthermore, given that 60% of human infectious diseases originate from zoonotic sources and that vectors are responsible for transmitting almost 20% of all such diseases, the role of VBDs is of significant concern [40]. The increasing incidence of these pathogens highlights a rising trend in outbreaks. This trajectory is illustrated in Figure 3, which outlines a timeline of the discovery and key milestones of emerging viruses in the 21st century.

Evidence indicates that a holistic approach, termed ‘One Health’, is imperative for achieving the overarching objective of optimizing public health outcomes. Human population growth, intensive farming and environmental degradation create interfaces where pathogens can cross-species boundaries. It is crucial to recognize that people, animals and ecosystems are interconnected to develop global surveillance and prevention strategies [11].

### 3.1. Ebolavirus Spillover

#### 3.1.1. Discovery and History

The Ebolavirus, which was discovered in 1976 near the Ebola River, in what is now the Democratic Republic of the Congo, represents one of the most feared transmissible pathogens globally [41]. The virus was first identified in a 42-year-old male who had purchased and consumed smoked antelope and monkey meat after a two-week road trip in northern Zaire [42]. The patient presented to a clinic on 26 August with chills and fever. The patient was treated for malaria, which provided temporary relief. A week later, the patient returned with severe symptoms. He died of an unknown cause on September 6 [43]. Since its initial discovery five decades ago, the virus has caused several outbreaks, primarily concentrated in Central and West Africa, affecting humans, chimpanzees, gorillas, and duikers [44,45]. Although these epidemics remained confined to small, isolated villages for decades, the devastating 2013–2016 outbreak, which resulted in almost 30,000 cases and over 11,000 recorded deaths, predominantly across Guinea, Liberia, and Sierra Leone, highlighted the critical need for effective medical countermeasures, significantly accelerating scientific research [44,46]. Since 2000, there has been an observed increase in the frequency of such outbreaks. This phenomenon has been correlated with an escalation in human–wildlife interactions, largely driven by deforestation and climate change, as indicated by studies conducted by Rojas and colleagues in 2020 [45,47].

#### 3.1.2. Taxonomy and Classification

Ebola virus is a linear, single-stranded, negative-sense RNA virus of about 19 kilobases. Its genome encodes seven structural proteins. The virus belongs to the order *Mononegavirales*, family *Filoviridae* and the genus *Ebolavirus*. The most deadly species is Zaire ebolavirus, which caused the 2013–2016 West African epidemic and subsequent outbreaks in the Democratic Republic of the Congo [48]. To date, six species of *Ebolavirus* have been identified. Of these, three are known to have caused major epidemics:

- *Ebolavirus* (EBOV), which causes Ebola virus disease (EVD);
- *Sudanvirus* (SUDV), which causes Sudan virus disease (SVD);
- *Bundibugyo* virus (BDBV), which causes Bundibugyo virus disease (BVD).

These three viruses are responsible for the majority of large outbreaks in Africa [49]. Ebola is the deadliest, and up to 90% of cases are fatal without treatment.

#### 3.1.3. Epidemiological Impact and Recent Outbreaks

The Ebola epidemic that occurred in West Africa from 2014 to 2016 was the most significant on record [50]. The virus exhibits a high degree of lethality, with a recorded mortality rate of approximately 50%. This has had a considerable effect on the region. The consequences of the virus were severe, with 28,000 infections and 11,300 deaths recorded, in addition to indirect fatalities due to other infections [51]. A minor Ebola outbreak occurred in the Democratic Republic of the Congo (DRC) in 2017, and a subsequent outbreak occurred in August 2018 [52]. The outbreak in Uganda, which was identified in September 2022, resulted in at least 55 fatalities before being declared over in January 2023 [53,54]. Notwithstanding the implementation of measures aimed at controlling epidemics, a considerable number of individuals residing in endemic regions remain susceptible to infection [55]. The Bundibugyo virus is the newest *Orthoebolavirus* known. First identified in 2007, it kills around 30% of infected people. The Democratic Republic of the Congo and Uganda have had two major outbreaks. In May 2026, an Ebola outbreak was confirmed in the Democratic Republic of the Congo and Uganda. On 2 June 2026, the WHO formally declared the 2026 Ebola outbreak in the DRC a global health emergency. There were 321 confirmed cases of the Bundibugyo strain of Ebola in the DRC, plus 116 suspected cases. There were 48 confirmed deaths and over 240 suspected deaths. Uganda has confirmed nine cases, one death and one suspected case [56].

#### 3.1.4. Transmission Pathways and Reservoirs

While the natural reservoir for all Ebola species has yet to be identified, numerous researchers consider fruit bats, particularly the *Rousettus aegyptiacus* species, to be the primary reservoir [44,45]. The virus is transmitted to humans through direct contact with infected individuals. In Africa, infections have been documented through the handling of sick or dead chimpanzees, gorillas, fruit bats, monkeys, forest antelopes, and porcupines [45]. Following spillover, the virus spreads within communities through person-to-person contact. Transmission can occur through direct contact with the blood, secretions, organs or other bodily fluids of infected individuals or through indirect contact with contaminated environments [44]. It has been established that traditional funeral practices involving corpse washing and direct handling play a significant role in the transmission of viruses. Furthermore, male survivors have been shown to be capable of perpetuating viral transmission through semen for up to 18 months after recovery [57].

#### 3.1.5. Pathogenesis and Immune Evasion

The Ebola virus’s remarkable lethal potential stems from its ability to initiate the early and comprehensive suppression of the host’s immune response. After entering the body, the virus infects cells belonging to the monocyte-macrophage lineage and dendritic cells. This target infection has been demonstrated to induce a blockade of molecular maturation, resulting in a progressive loss of antigen-presentation capacity and an inefficient activation of helper and cytotoxic T lymphocytes. Concurrently, the viral proteins VP35 and VP24 suppress the phosphorylation and nuclear translocation of the transcription factors IRF3 and STAT1, thus repressing the host cell’s production of type I interferons. The neutralization of the adaptive immune compartment results in the aberrant triggering of a massive secretion of pro-inflammatory mediators by infected macrophages, leading to a cytokine storm [58]. This hyperinflammatory state has been shown to lead to a significant increase in endothelial permeability and compromise of vascular barrier integrity, often resulting in a condition known as disseminated intravascular coagulation [59].

#### 3.1.6. Clinical Manifestations and Diagnosis

Ebola virus disease is a severe illness that manifests with fever, weakness, muscle pain, headache, and throat pain. These symptoms may be accompanied by vomiting, diarrhea, rash, kidney and liver problems, and, in some cases, bleeding. The incubation period is reported to range from 2 to 21 days [41]. Laboratory findings often show leukopenia, thrombocytopenia and elevated liver enzymes. For decades, Ebola diagnosis relied on conventional methods such as viral isolation and ELISA for specific antibodies. However, molecular assays using polymerase chain reaction (PCR) are now the gold standard for detecting viral RNA during the acute phase of infection [44].

#### 3.1.7. Vaccination and Containment

The clinical management of individuals with suspected or confirmed contact has benefited significantly from the introduction of treatment protocols and post-exposure prophylaxis utilizing antiviral agents and immunotherapies, which are administered either intramuscularly or intravenously. These approaches have been demonstrated to mitigate disease severity, curtail viral replication kinetics and reduce mortality rates [46]. The WHO strongly recommends treatment with mAb114 (ansuvimab) or REGN-EB3 (Inmazeb), two monoclonal antibody therapies [60]. In the context of vaccine development, two vaccines have been authorized for deployment against the Ebola virus. However, neither of these vaccines has been approved for use in cases of infection with the Bundibugyo virus. Ervebo (Merck & Co., Rahway, NJ, USA) and the two-dose regimen Zabdeno and Mvabea (Janssen Pharmaceutica, Beerse, Belgium) can be used to limit the spread of the Zaire ebolavirus through a so-called ring vaccination strategy [61]. Upon confirmation of a case, the administration of the vaccine to all close contacts and contacts-of-contacts establishes an immunological barrier that effectively “freezes” and geographically circumscribes the outbreak [44]. Community engagement is key to containing outbreaks. Public awareness campaigns are key to preventing zoonotic transmission and include avoiding bats or monkeys, refraining from raw bushmeat, and rapidly isolating patients. Healthcare workers must follow rigorous infection control measures to prevent contact with bodily fluids and surfaces [62]. According to the models’ predictions, spillover intensity is maximized in regions experiencing forest loss, marked by substantial shifts in the human population and notable alterations in meteorological conditions [18,63,64].

### 3.2. West Nile Spillover

West Nile virus (WNV) is an arthropod-borne virus (arbovirus) belonging to the *Flaviviridae* family. It is an enveloped, positive-stranded, ribonucleic acid (RNA) virus. First isolated in 1937 in the West Nile district of Uganda, this virus is the most geographically widespread arbovirus, being present in Africa, Western Asia, Europe, Australia and the Americas [65]. The virus is transmitted among avian species by infected mosquitoes (especially of the *Culex* genus, which act as the natural reservoir). Furthermore, mosquito bites represent the principal route of human transmission of the virus, with no evidence of human-to-human transmission of WNV through contact with an infected individual. Although less prevalent, other documented routes include blood transfusions and mother-to-child transmission during pregnancy [66]. The virus has the capacity to induce an infectious condition in humans, ranging from asymptomatic infection to severe neurological disease, including meningitis, encephalitis and flaccid paralysis [67]. Although it has been established that human infection does not yield sufficient viremia to infect mosquitoes, meaning that outbreaks are limited to spillover events from the wildlife cycle, the global impact of WNV is still significant, causing thousands of deaths [68]. The virus is endemic in Africa but is spreading yearly through the Western Hemisphere via migrating birds. Climate change is impacting mosquito breeding and bird migration, creating more opportunities for the virus to spread [69]. Human activities may be increasing contact between vectors and hosts. The virus is maintained in birds and mosquitoes, with humans acting as dead-end hosts. This underscores the unidirectional nature of the interaction [70]. Public health responses to the virus should include the establishment of surveillance systems, the monitoring of bird deaths, mosquito pools and human cases. As no vaccines are currently available for human use, prevention through mosquito control and personal protection is of the utmost importance [70].

### 3.3. SARS-CoV-1, SARS-CoV-2, and MERS-CoV Spillover

#### 3.3.1. Classification of Coronaviruses

SARS-CoV-2, SARS-CoV, and MERS-CoV belong to the *Betacoronavirus* genus within the *Coronaviridae* family [71]. These enveloped, single-stranded RNA viruses are known for causing severe respiratory illnesses in humans. SARS-CoV, MERS-CoV and SARS-CoV-2, the causative agents of SARS, MERS and COVID-19, represent the three major, highly pathogenic outbreaks of coronaviruses that have spilled over from animal reservoirs to infect humans in the 21st century [72]. Although all three pathogens originated in bats, they have followed different pathways to reach human populations, resulting in unique clinical and transmission characteristics [73]. Coronaviruses have traditionally been regarded as causative agents of only mild respiratory infections in humans. However, the emergence of the SARS (severe acute respiratory syndrome) outbreak in China in 2002–03, caused by a virus designated SARS-CoV, shifted this paradigm [74]. The SARS-CoV outbreak exhibited a 9% mortality rate, which increased to 50% in individuals above the age of 60, thereby classifying it as the most severe disease caused by a coronavirus [75]. Although the scientific literature identifies horseshoe bats as the natural reservoir of the SARS-CoV virus, the precise path of the zoonotic spillover remains under investigation. Following the 2002–2003 outbreak, epidemiological and phylogenetic investigations identified SARS-like coronaviruses circulating in bats of the genus *Rhinolophus* as the natural reservoir [76]. Comparative immunology studies revealed remarkable evolutionary adaptations in bats, including modulation of the STING (stimulator of interferon genes) signaling pathway and constitutive yet tightly controlled expression of type I interferons [77]. This immunological profile limits cytopathic viral replication while preventing excessive inflammatory responses, thereby allowing viral persistence without overt clinical disease [77].

#### 3.3.2. Intermediate Hosts and Management of SARS-CoV-1

The spillover of SARS-CoV-1 into humans was not direct but was mediated by intermediate amplification hosts, primarily the masked palm civet (*Paguma larvata*) and the raccoon dog (*Nyctereutes procyonoides*) [78]. These species were frequently kept under overcrowded and stressful conditions in live animal markets, which promoted immunosuppression and enhanced viral shedding through respiratory and fecal secretions. Such environments functioned as evolutionary bioreactors, increasing interspecies contact and thus facilitating viral adaptation [79]. SARS-CoV-1 crossed the species barrier due to key stereochemical modifications in the spike glycoprotein. The S1 subunit contains the receptor-binding domain (RBD), which is responsible for interacting with the host receptor, angiotensin-converting enzyme 2 (ACE2). Binding studies have shown that bat-adapted viral variants have limited affinity for human ACE2 receptors [80]. During circulation in intermediate hosts, adaptive point mutations within the RBD—particularly at amino acid positions 479 and 487—enhanced interactions with Lys31 and Lys353 residues of human ACE2 through hydrogen bonding and van der Waals forces. These adaptations enabled efficient attachment of the Spike protein to respiratory epithelial cells, facilitating membrane fusion and viral entry [81]. Retrospective analyses performed within the One Health framework indicate that the emergence of SARS-CoV-1 was largely driven by anthropogenic factors [82]. The increasing demand for animal protein, coupled with the unregulated wildlife trade, has disrupted the ecological barriers that have historically prevented bat-associated viruses from spreading to human populations [83].

The scientific literature highlights a significant shortcoming in the management of the outbreak: the response was largely reactive and anthropocentric, with a focus on hospital containment and patient isolation. There was a lack of integrated veterinary and environmental surveillance systems. Following the apparent eradication of the virus and the depopulation of the market, investment in the active surveillance of *Sarbecoviruses* declined. This left unresolved ecological vulnerabilities that would later contribute to the emergence of SARS-CoV-2 [8]. The outbreak was largely contained because the transmission of SARS-CoV was relatively inefficient, occurring exclusively through direct contact with an infected individual [84].

#### 3.3.3. MERS-CoV Epidemiology, Reservoirs, and Transmission

In 2012, the Middle East witnessed an outbreak of severe respiratory tract infections, attributable to a novel type of coronavirus, designated MERS-CoV (Middle East respiratory syndrome) [85]. MERS-CoV is classified within the genus *Betacoronavirus* and the subgenus *Merbecovirus.* The disease is characterized by exceptionally high clinical severity, with an estimated case fatality rate of around 35%, which is substantially higher than that observed for other known human coronaviruses. As with SARS-CoV-1 and SARS-CoV-2, it is believed that MERS-CoV is bat-borne [35]. However, unlike *sarbecoviruses*, which exhibit different transmission patterns, spillover in this instance was attributed to prolonged and systematic interaction with a domestic reservoir host rather than sporadic exposure to wildlife [86]. The dromedary camel (*Camelus dromedarius*) is the main host of MERS-CoV, rather than just an intermediate host [87]. Serological studies have demonstrated the circulation of MERS-CoV in dromedary populations across the Arabian Peninsula and sub-Saharan Africa for decades prior to the first recognized human outbreak. In dromedaries, infection is largely restricted to the upper respiratory tract and generally produces mild clinical signs such as rhinitis, particularly in animals younger than two years of age. During infection, large quantities of virus are shed through nasal and ocular secretions, representing the principal source of human exposure. Transmission occurs through close contact during animal husbandry, milking, slaughtering activities, or through short-range aerosol exposure [88]. MERS-CoV achieved zoonotic transmission through interaction between the receptor-binding domain of the Spike protein and dipeptidyl peptidase 4 (DPP4/CD26), a receptor expressed on respiratory and renal epithelial cells. Unlike ACE2, DPP4 is highly conserved across species, and the camel and human receptors exhibit remarkable structural similarity, facilitating cross-species infection [89]. The epidemiological profile of MERS-CoV is characterized by a marked asymmetry between spillover and human-to-human transmission. Although highly efficient at infecting humans from camels, the virus exhibits limited transmission efficiency within human communities. Sustained transmission chains are therefore uncommon and generally occur only in healthcare settings [87].

Disease control cannot rely solely on clinical management of human cases but must focus on the animal–human interface [90]. Veterinary vaccination programs employing viral vectors that express the MERS-CoV Spike protein are among the current strategies in use, with the aim of reducing viral shedding in young dromedaries and thus interrupting transmission at its origin. Further measures have been proposed, which include the implementation of improved slaughterhouse regulations, public education regarding the consumption of raw camel milk and undercooked meat, and the introduction of integrated transboundary surveillance programs. This is particularly relevant given the large-scale movement of dromedaries between the Horn of Africa and the Arabian Peninsula [90,91]. As stated in the WHO report, by the close of January 2020 the MERS-CoV virus had disseminated to 27 countries. The report also stated that a total of 2519 laboratory-confirmed cases of MERS had been recorded, including 866 associated deaths [92].

#### 3.3.4. The Spread of SARS-CoV-2

Numerous studies have demonstrated that the virus responsible for Middle East respiratory syndrome (MERS)-coronavirus (CoV) has its origins in bats, in a manner analogous to that of severe acute respiratory syndrome (SARS)-coronavirus. Furthermore, the etiology of MERS-CoV is believed to originate in bats and subsequently disseminate to camels, which are considered the primary reservoir for human infection. In December 2019, a group of patients suffering from pneumonia of unknown origin was reported. The initial cases were found to be significantly associated with the Huanan Seafood Wholesale Market, which is considered to be the point of origin for the spillover of the virus from animals to humans. This outbreak marked the emergence of severe acute respiratory syndrome coronavirus 2 (SARS-CoV-2), which subsequently led to the global propagation of the pandemic that has been designated ‘COVID-19′ [93].

SARS-CoV-2 belongs to the subgenus *Sarbecovirus* and shares approximately 79% genomic sequence identity with SARS-CoV-1. It is the causative agent of COVID-19, the disease responsible for the most significant global health crisis of the twenty-first century [82]. Despite its genetic relatedness to SARS-CoV-1, SARS-CoV-2 displayed markedly different transmission dynamics and pathogenic characteristics. The hierarchical barriers model provides a useful framework for understanding SARS-CoV-2 spillover. According to this model, a virus must overcome multiple ecological and biological barriers simultaneously: ecological barriers through increased human–bat overlap associated with land-use change; physiological barriers through extensive asymptomatic viral shedding; molecular barriers through the presence of a furin cleavage site and high affinity for ACE2; and immune barriers through efficient evasion of early type I interferon responses. From the earliest stages of the outbreak, SARS-CoV-2 demonstrated exceptional capacity to overcome these barriers, exhibiting high replicative fitness and efficient human-to-human transmission, including transmission from asymptomatic individuals [94].

#### 3.3.5. Virological Features and Reverse Zoonotic Transmission of SARS-CoV-2

The most distinctive virological feature of SARS-CoV-2 is the presence of a polybasic furin cleavage site at the S1/S2 junction of the Spike protein, characterized by the amino acid insertion PRRAR. Because furin is ubiquitously expressed in human tissues, particularly within the respiratory tract, Spike proteins can be pre-activated during viral egress. Consequently, newly released virions are already primed for membrane fusion, expanding tissue tropism, increasing infectivity, and promoting elevated viral loads in the upper respiratory tract, a key driver of aerosol transmission. Potential ecological drivers of spillover include deforestation, agricultural expansion, and uncontrolled urbanization within habitats occupied by Rhinolophus bats [95]. These processes increase nutritional stress and immune dysregulation in bat populations, favoring viral reactivation and shedding. Habitat fragmentation also promotes contact among bats, livestock, domestic animals, and humans through contaminated food resources and shared environments, thereby increasing pathogen pressure and the likelihood of spillover [94]. The evolutionary success of SARS-CoV-2 in humans should also be interpreted in the context of spillback, or reverse zoonosis. Following adaptation to humans, the virus spread to numerous domestic, synanthropic and wild animal species. Intensive mink farming provided a notable example: infected workers transmitted the virus to mink populations, where rapid circulation favored the accumulation of adaptive Spike mutations. Subsequent transmission back to humans demonstrated the potential for bidirectional zoonotic cycles and altered susceptibility to neutralizing antibodies [96]. Another species significantly affected by SARS-CoV-2 is the white-tailed deer (*Odocoileus virginianus*). In these populations, the virus has become established within wildlife, creating a secondary reservoir. The persistence of SARS-CoV-2 in wild animal populations raises concerns regarding the emergence of cryptic variants capable of re-entering the human population and potentially reducing vaccine effectiveness [97]. The SARS-CoV-1 and SARS-CoV-2 pandemics clearly demonstrated that the traditional separation between human and animal health is no longer sustainable. Effective One Health strategies must move beyond post-spillover clinical responses and prioritize primary prevention through sustainable land-use planning, reduction in habitat fragmentation, protection of ecological hotspots, and continuous surveillance of viruses circulating in wildlife, livestock, and companion animals. Additional measures include restructuring live animal markets and strengthening biosecurity protocols in susceptible animal production systems [97].

### 3.4. Insights into Zika Virus Pathogenesis, Spillover Prevention, and Surveillance

#### 3.4.1. Zika Virus Transmission Cycle

The Zika virus (ZIKV) is characterized by a positive-sense, single-stranded RNA genome that is encapsulated within an icosahedral, enveloped lipid structure. It is classified within the *Flaviviridae* family and the *Orthoflavivirus* genus (formerly classified as *Flavivirus*). Other noteworthy members of this family include the dengue virus (DENV), the WNV and the yellow fever virus (YFV) [98]. Although all of these flaviviruses are classified as arthropod-borne viruses (arboviruses), they exhibit significant variation in terms of their specific clinical impacts and geographic distribution. The virus was first isolated in 1947 from a sentinel *Rhesus macaque* in the Zika Forest of Uganda. Subsequently, in 1948, it was identified in *Aedes africanus*, a mosquito species indigenous to the same area. The first documented instance of the disease in a human occurred in Nigeria in 1954. The virus initially manifested as sporadic cases; however, it subsequently caused major outbreaks, including an epidemic in 2007 on the Western Pacific island of Yap in the Federated States of Micronesia. This was followed by a larger epidemic in French Polynesia in the South Pacific during 2013 and 2014, which resulted in an estimated 30,000 symptomatic infections [99]. ZIKV is primarily transmitted to humans through the bite of an infected arthropod vector. However, other documented routes of transmission include mother-to-child, blood transfusion, bone marrow or organ transplantation, and sexual contact. The ZIKV displays two distinct transmission cycles. The former is referred to as the enzootic or sylvatic cycle, and it is prevalent in natural environments. The second type, designated as the epidemic or urban cycle, is predominant among urban populations and occurs in human-made environments. In both cycles, the virus is primarily transmitted by mosquitoes of the genus *Aedes*; however, secondary routes of non-vector-borne transmission have been shown to have a significant impact on the urban cycle [100]. The sylvatic cycle involves the virus’s natural circulation between canopy-dwelling mosquitoes (e.g., *Aedes* species) and non-human primates (e.g., monkeys). The urban cycle follows: after a spillover event, the virus transitions into a human-to-mosquito-to-human cycle. Peridomestic mosquitoes like *Aedes aegypti* and *Aedes albopictus* thrive in urban environments and are the primary vectors [101].

#### 3.4.2. Neurological Complications of Zika Virus Infection

This flavivirus, which originated on the African continent and then migrated to Southeast Asia, has been shown to cause maternal infection and viral transmission to the fetus. Infection of neural progenitor cells of the fetal brain by ZIKV can result in microcephaly, defined by an infant’s head size that is smaller than expected for their age and gender, accompanied by impaired neurological development [101]. Briefly, following initial exposure, the virus primarily infects skin cells, such as keratinocytes and fibroblasts, through the interaction between viral surface proteins and host AXL cellular receptors. Following internalization within human cells, the virus undergoes uncoating and initiates replication of its RNA genome. The infected cells then migrate to regional lymph nodes, facilitating viral entry into the bloodstream. ZIKV demonstrates a robust neurotropism, enabling it to traverse both the blood–brain barrier and the placental barrier. Upon reaching the fetus, the virus targets fetal neural progenitor cells (NPCs), inducing cell cycle arrest and apoptosis [102]. In 2015, the Brazilian Ministry of Health definitively established a correlation between Zika virus infection and teratogenic outcomes. Specifically, fetuses infected congenitally by mothers presented with microcephaly, a congenital malformation characterized by an underdeveloped head circumference [102]. According to prevailing clinical guidelines, the standard measurement for newborn head circumference is 31.9 cm for males and 31.5 cm for females. However, in 2016 alone, 7343 cases of microcephaly were documented in Brazil. Subsequent studies have demonstrated that the probability of developing microcephaly increases significantly if ZIKV exposure occurs during the first trimester of pregnancy, a critical window coinciding with fetal organogenesis [103]. In contrast, in adults, ZIKV infection generally manifests with non-specific, mild symptoms such as fever, cutaneous rash, arthralgia, myalgia, gastrointestinal distress, and general malaise. These symptoms generally resolve without pharmacological intervention within a few days to a week. Nevertheless, sporadic cases have been linked to Guillain-Barré syndrome (GBS), an autoimmune disorder in which the immune system aberrantly attacks the myelin sheath and axons of peripheral nerves, leading to progressive paralysis. To date, no specific antiviral treatment is available, and there is currently no commercially approved vaccine against the Zika virus [101].

#### 3.4.3. Zika Virus Detection, Surveillance and Control

With regard to diagnostic strategies, molecular assays based on polymerase chain reaction (PCR) have been approved and are established as the gold standard for detecting viral RNA during the acute phase of infection. These molecular tests are primarily performed on whole blood, serum, and urine specimens; notably, urine samples are highly recommended as they often exhibit higher viral loads and offer a wider diagnostic window compared to blood components. Conversely, serological assays are not widely recommended for primary diagnosis due to significant limitations. In the initial phase of acute infection, the presence of specific IgM antibodies is often undetectable. Additionally, these tests are highly susceptible to false-positive results resulting from antibody cross-reactivity with other co-circulating flaviviruses, such as the dengue virus [104]. Consequently, the risk of viral spillover remains high in low- and middle-income countries characterized by high population density and inadequate sanitation, particularly across Asia and Africa. Prevention remains the primary line of defense, relying on personal protective measures—such as the use of insect repellents and bed nets to prevent mosquito bites in endemic areas—alongside vector control programs to suppress arthropod proliferation. Robust epidemiological surveillance is imperative to monitor case counts and detect potential outbreaks early, alongside public health campaigns to raise awareness, particularly among pregnant women [105]. In order to address these global health priorities on a worldwide basis, the WHO has established the Global Outbreak Alert and Response Network (GOARN). This global alliance of institutions functions as a collaborative network designed to signal, confirm, and identify emerging public health alerts as well as epidemic hotspots [106].

### 3.5. Nipah Virus Genotypes: Transmission Pathways, Pathogenesis and One Health Strategies

#### 3.5.1. NiV Genome, Transmission Dynamics and Strain Differences

The Nipah virus (NiV) is an enveloped, single-stranded, negative-sense RNA virus belonging to the genus *Henipavirus* within the *Paramyxoviridae* family. Fruit bats of the genus *Pteropus* act as the natural reservoir for the virus. The WHO has classified the Nipah virus (NiV) as one of the most lethal pathogens known, with a case fatality rate ranging from 40% to 90%, depending on the viral genotype [107]. The Centers for Disease Control and Prevention (CDC) has classified Niv as a Category C pathogen, which encompasses emerging pathogens with the potential to be engineered for mass dissemination as a biological threat. The virus was first identified during the 1998 outbreak in Malaysia, which resulted in 265 human cases and 105 deaths [107]. The spillover of NiV from bats to an intermediate amplifier host, specifically pigs, occurred via environmental contamination. In contrast to the asymptomatically infected reservoir hosts, pigs exhibit symptoms of severe acute respiratory syndrome and actively excrete the virus to humans. Consequently, the primary cohort affected was that of swine-farm workers, due to their direct contact with infected animal bodily fluids [108]. Furthermore, alternative transmission pathways comprise the ingestion of food contaminated with bat excreta and subsequent human-to-human transmission via direct contact with infectious bodily fluids. Following an incubation period which can vary between four and fourteen days, the virus attaches to host cell-surface-displayed ephrin-B2 or -B3 receptors located on endothelial and neuronal cells. This binding represents the initiation of the virus’s replication process, which subsequently leads to the release of viral progeny [109,110]. Since 2001, outbreaks of NiV have occurred annually, predominantly during the winter months from December to March, in Bangladesh. Phylogenetic analyses indeed confirm that NiV splits into two major genetic lineages: the Malaysia strain (NiV-M) and the Bangladesh strain (NiV-B) [111]. Despite the fact that these strains exhibit 91.8% nucleotide identity, they are responsible for outbreaks in different geographical areas, exhibiting notable differences in their transmission dynamics, fatality rates, incubation periods, and clinical manifestations. Although the clinical manifestation of the illness usually commences with the onset of a respiratory syndrome, characterized by fever, cough and headache, differences in transmission patterns and mortality rates suggest that NiVB may be more pathogenic than NiVM. Recent studies have indicated that NiVB outbreaks have a considerably higher case mortality rate (CFR) of approximately 75%, in contrast to the 40% observed in NiVM outbreaks [112]. Furthermore, NiV-M is principally a zoonotic threat with limited human-to-human transmission, unlike the hallmark of NiVB. The NiVB has been observed to target the brain and the respiratory tract, often presenting as atypical pneumonia and acute respiratory distress. NiV-M has no seasonal pattern, relying on pigs as intermediate hosts and showing limited human-to-human transmissibility. In contrast, NiV-B sticks to winter, circumventing the need for an intermediate host. It is often transmitted via the consumption of contaminated date sap, demonstrating robust person-to-person transmission and a mortality rate of approximately 70% [107,113]. Furthermore, human-to-human transmission is driven by nosocomial pathways. In such settings, healthcare workers and caregivers are exposed to a higher risk of infection due to inadequate initial isolation protocols, which are often the result of limited resources [107].

#### 3.5.2. Prevention Strategies Within the “One Health” Approach

The gold standard for NiV laboratory diagnosis is quantitative Reverse Transcription Polymerase Chain Reaction (RT-PCR), a method employed to detect viral RNA in nasopharyngeal/throat swabs, cerebrospinal fluid (CSF), or whole blood. In the later stages of infection, enzyme-linked immunosorbent assays (ELISA) are utilized for the detection of IgM and IgG antibodies. These antibodies represent a significant asset in the context of seroepidemiological studies conducted on affected populations. Nevertheless, an accurate diagnosis requires highly skilled professionals and advanced containment facilities, which are frequently not available in regions facing resource constraints and widespread disease [114]. Given that no specific antiviral therapy or vaccine has been approved to date, clinical management is limited to the administration of strict measures to support patients, with the aim of alleviating respiratory and neurological symptoms. Consequently, the containment of outbreaks is contingent on the implementation of preventive measures, including the avoidance of contact with sick animals, thorough fruit washing, and the maintenance of rigorous hand hygiene. It is imperative that healthcare workers strictly adhere to personal protective equipment protocols, and that laboratory handling is conducted in accordance with Biosafety Level 4 (BSL-4) containment protocols [115]. The WHO South-East Asia Region (SEAR) Roadmap Security (2023–2027) promotes the adoption of a multidisciplinary “One Health” approach. This strategy integrates human, animal, and environmental surveillance, for example by monitoring the circulation of viruses in bat populations near livestock farms. This approach addresses the key drivers of deforestation and agricultural expansion, which have been identified as key factors in forcing reservoir hosts into closer proximity with livestock and human settlements [95,116,117].

### 3.6. Spillover of Influenza A Virus: A Continuous Threat to Public Health

#### 3.6.1. Orthomyxoviridae: Structure and Impact

The *Orthomyxoviridae* family is currently divided into four genera: *Alphainfluenzavirus* (influenza A virus), *Betainfluenzavirus* (influenza B virus), *Gammainfluenzavirus* (influenza C virus), and *Deltainfluenzavirus* (influenza D virus). Among these, the genera *Alphainfluenzavirus* and *Betainfluenzavirus* represent the primary etiological agents of human seasonal epidemics. In contrast, the genus *Gammainfluenzavirus* has historically been linked to mild or paucisymptomatic clinical manifestations in both pediatric and adult populations. Conversely, the genus *Deltainfluenzavirus* circulates predominantly among livestock reservoirs (specifically cattle and swine), and its zoonotic potential remains under active investigation [118,119].

Influenza A virus (IAV) is an enveloped, single-stranded, negative-sense RNA virus with a lipid envelope. It has eight gene segments (nine for the IAV genome of influenza C). The eight segments encode various proteins, typically 10, including PB2, PB1, polymerase acidic protein (PA), hemagglutinin (HA), and others. This segmented architecture is of critical evolutionary importance, as it enables the process of genetic reassortment (antigenic shift), whereby gene segments from different viruses combine when infecting the same host cell, resulting in the formation of a novel hybrid virus [120]. The pandemic strain that emerged in 2009 has been shown to be the result of a complex quadruple genetic reassortment, which combined genetic material from different animal reservoirs. The main natural reservoir of influenza A viruses is wild waterfowl. These are mainly ducks, geese and swans, gulls, terns and waders [121]. Over thousands of years, the virus has evolved to function in a state of perfect parasitism within these species. The infection is mostly asymptomatic, with viral replication mainly in the gastrointestinal tract. As a result, a significant quantity of virions is shed into the environment via feces. Cross-species transmission is primarily governed by the specificity of hemagglutinin (HA) in binding sialic acid (SA) receptors. Avian viruses recognize sialic acid linked to galactose via an -2,3-linkage, predominant in the avian intestines. Human viruses exhibit a selective tropism for the -2,6-linkage, predominant in the human upper respiratory tract. Humans have -2,3 receptors within the deep respiratory tract, specifically at the alveolar level [122]. If an individual comes into contact with infected birds (in intensive farming), deep inhalation of the virus can trigger a spillover event by overcoming the species barrier.

#### 3.6.2. Genomic Architecture and Cross-Species Transmission Mechanisms

The most perilous route for the initiation of a pandemic event is through the involvement of the swine host, as it is the only species that expresses both -2,3 and -2,6 receptors on its respiratory mucosa. In the event of a pig being infected with different types of influenza, the pathogens’ RNA replicates in the same cell nucleus and is exported to the cytoplasm. This process gives rise to a novel type of virus with the capacity to propagate among human hosts. The ease with which the virus spreads is due to its use of the -2,6 receptors, against which the human population has no immunity. This allows the reassorted virus to undergo spillover into the human host, not only infecting them but also rendering them capable of efficient human-to-human transmission via aerosols, thereby laying the groundwork for a new pandemic—as occurred in 2009 with the A(H1N1) pdm09 strain [123]. The annals of public health are characterized by recurrent pandemics, initiated by antigenic shifts in these viruses. Notable examples include the 1918 “Spanish” flu (H1N1), the 1957 “Asian” flu (H2N2), and the 1968 “Hong Kong” flu (H3N2). In 2009, with the emergence of the pandemic strain A(H1N1) pdm09, a redefined paradigm for virological surveillance was established. It was highlighted that the virus had the capacity to circulate silently in animal reservoirs for decades before achieving definitive spillover into the human population [124]. IAVs are a constant, continuously evolving global health threat. They cause a significant epidemiological and socio-economic burden, with seasonal influenza causing between 3 and 5 million cases of severe illness and 290,000–650,000 respiratory deaths each year. All age groups are vulnerable to infection but children under five, pregnant women, the elderly and those with compromised immune systems are most at risk. Epidemiological studies have shown that seasonal influenza viruses often acquire mutations that alter their replication and antigenic profiles [124]. Recent seasonal H1N1 viruses, for instance, have acquired mutations in the receptor-binding site (RBS) of the hemagglutinin (HA) protein and within antigenic sites, branching into clades 5a.2a and 5a.2a.1. This continuous genetic remodeling underscores the importance of studying original spillover events and pathogenic mechanisms [125].

#### 3.6.3. Viral Pathogenesis and Immune Evasion

Human infection begins with inhaling aerosols or droplets containing viral particles. The viral HA binds to sialic acid residues on epithelial cells in the upper respiratory tract. The virus enters via receptor-mediated endocytosis and begins to replicate, initially showing a tropism for the ciliated cells of the tracheobronchial respiratory tree. As the virus replicates, it causes the host epithelial cells to lyse, resulting in desquamation of the respiratory epithelium. This damage is responsible for local symptoms such as dry cough and susceptibility to secondary bacterial superinfections due to the loss of mucosal barrier integrity [126]. At the intracellular level, the virus is recognized by the host’s innate immunity receptors (e.g., TLR3, TLR7 and RIG-I), which trigger the production of pro-inflammatory cytokines and type I interferon (IFN). However, IAV expresses the non-structural protein NS1, a potent virulence factor that antagonizes IFN signaling pathways, thereby delaying the innate immune response during the early stages of infection and permitting extensive viral replication. From a clinical standpoint, the infection typically manifests with acute symptoms. Although the course is often self-limiting, it can progress to severe clinical presentations in fragile or high-risk individuals [126]. In severe cases or those caused by highly virulent strains, the initial delay in the immune response and the high viral load lead to a massive, dysregulated recruitment of immune cells into the pulmonary compartment. This exaggerated influx provokes an overactivation of pro-inflammatory cytokine production, resulting in acute damage to the pulmonary vascular endothelium. This can progress to viral sepsis and multiple organ dysfunction [127].

#### 3.6.4. Diagnosis and Prevention

Timely diagnostic protocols and constantly updated immunological prevention strategies are key to managing and containing the spread of the influenza virus. The gold standard for identifying and typing viruses is molecular nucleic acid amplification tests, specifically RT-PCR (Reverse Transcription Polymerase Chain Reaction) performed on nasopharyngeal swabs or lower respiratory tract samples. Molecular diagnostics allow for rapid infection confirmation, viral subtyping and gene sequencing. These critical steps help to monitor resistance mutations against first-line antiviral drugs. In viral sepsis and intensive care, molecular monitoring can also be extended to blood samples to intercept early cases of viremia, thereby preventing systemic spillover and organ damage. Annual vaccination is the most effective way to reduce the incidence of severe forms and hospitalization. The WHO updates seasonal trivalent and quadrivalent influenza vaccines biannually due to the continuous antigenic drift of the virus and the stabilization of new genetic clades. The vaccine includes antigens engineered to protect against currently circulating strains, paired with biosecurity measures in swine and poultry farms [128].

### 3.7. Routes of MPXV Spillover

#### 3.7.1. Morphology and Transmission Pathways of MPXV

MPXV is a DNA virus belonging to the genus *Orthopoxvirus* and the family *Poxviridae*. It is oval and 200–250 nm wide, with a double-stranded DNA genome. It is a zoonosis transmitted via rodents, with humans acting as susceptible hosts. Transmission occurs via contact with infected blood or bodily fluids, direct contact with infected skin, mucous membranes or lesions and bites from infected animals. The infection is also transmitted from one human to another via respiratory droplets or during sexual transmission [129].

#### 3.7.2. Discovery and History

The monkeypox virus was first isolated at the State Serum Institute in 1958. Its name stems from the monkeys it was first observed in, though these are not its natural reservoir. It primarily circulates in African rodents and mammals. Between 1960 and 1968, sporadic cases were identified in U.S. captive monkey communities. The first documented case of human infection occurred in 1970 in the Democratic Republic of the Congo. The patient exhibited symptoms consistent with smallpox and had not been vaccinated [130]. Additional cases were documented among infants in Liberia and Sierra Leone. The virus prevalence was notably high in Africa, where it was considered an endemic disease in the region.

#### 3.7.3. International Outbreaks and the Global Pandemic

The first outbreak outside of Africa was in the United States in 2003. It started with an animal shipment from Ghana containing MPXV-infected rodents. These rodents transmitted the virus to captive prairie dogs intended for trade. This led to 47 confirmed human cases. In 2022, MPXV expanded rapidly across multiple continents, including Europe, North America, South America and Asia. By the end of July 2024, the global outbreak had resulted in 102,997 confirmed cases, 186 probable cases and 223 deaths [131,132].

The WHO declared Mpox a Public Health Emergency of International Concern (PHEIC). The countries with the highest incidence of infection since 1 January 2022, were the United States (33,556 cases), Brazil (11,841 cases), Spain (8104 cases), the Democratic Republic of the Congo (4385 cases), France (4283 cases), Colombia (4256 cases), Mexico (4132 cases), the United Kingdom (4018 cases), Peru (3939 cases), and Germany (3886 cases) [133].

#### 3.7.4. Viral Clades and Lineages

Two distinct MPXV clades have been identified. Clade I is historically associated with more severe clinical manifestations and elevated mortality rates. Clade II generally results in reduced disease severity and lower fatality rates. Subsequent to the primary classification, each clade undergoes further subdivisions, resulting in the identification of specific subclades. Of particular relevance, subclades IIa and IIb exhibit an enhanced propensity for interhuman transmission [134]. The 2022 global multi-country outbreak was driven by an MPXV lineage nested within Clade IIb. This outbreak was subsequently designated as clade hMPOXV, characterized by specific cytosine-to-thymine (C → T) or guanine-to-adenine (G → A) transitions [135].

#### 3.7.5. Symptoms, Diagnosis, Treatment and Spread

The incubation period of MPXV is estimated to range from five to 21 days. Most fatalities have been observed among immunocompromised individuals, pregnant women, and children. The infectious form of MPXV that originates from Clade I shares strong similarities with smallpox. However, lymphadenopathy is a distinctive characteristic of Mpox that serves as a hallmark clinical indication of the disease [136].

MPXV demonstrates tissue tropism at various sites, including the mouth, genitals, cornea and gut. PCR is the gold standard for viral diagnosis. Clinical samples for confirmation include skin scrapings from rashes, crusts, or fluid from blisters. Other diagnostics include immunohistochemistry and electron microscopy [137]. Tecovirimat is the primary antiviral used for severe Mpox treatment. It works by inhibiting the viral envelope protein VP37, which is critical for releasing mature viral particles. Brincidofovir represents an additional oral antiviral alternative. As a cidofovir lipid conjugate, it impedes MPXV DNA replication. However, adverse side effects limit its clinical use. Experimental findings indicate that brincidofovir and smallpox vaccination may together reduce the severity of cutaneous lesions [138]. The transmission of this pathogen is promoted by a multifactorial matrix of environmental, intrinsic, and host factors that collectively drive the dissemination of MPXV between natural reservoirs and susceptible hosts [139]. The primary drivers implicated in this process are categorized below:

- Environmental Resistance and Stability

Like other *Orthopoxvirus* genus members, MPXV can persist on surfaces and in environments for weeks, highlighting its ability to remain infectious in ambient conditions for days [140].

b. Intrinsic Viral Factors and Accelerated Mutation

*Orthopoxviruses* have stable double-stranded DNA genomes and low mutation rates due to polymerase proofreading. However, the 2022 outbreak exhibited a higher than predicted mutation rate. Phylogenetic analyses traced these genetic modifications back to endemic strains in West Africa from 2017 to 2019. The accelerated microevolution is likely driven by human APOBEC3-mediated editing, enhancing the virus’s adaptation to human-to-human transmission [141].

c. Host-Related Factors and Transmission Changes

In the past, active pustules and crusts were the main source of infection, as it was thought the period of transmission ended when the lesion was fully covered. But cases without a classic rash show that transmission is not just from direct contact of lesions. Evidence highlights the importance of alternative transmission routes, like respiratory droplets. Viral DNA has been found in the upper respiratory tract for weeks after infection [142]. Sexual contact was the main driver of the 2022 global epidemic. Unlike previous African endemic cases, where rashes were widespread on the face and limbs, the 2022 cases showed oligolesions in the genital, perineal, or perianal area. MPXV DNA has been identified in a variety of biological specimens, including saliva, rectal swabs, nasal and throat swabs, urine, feces, and semen. It has also been found in cell culture, proving it can be infectious [33].

d. Non-Canonical Transmission Routes and Novel Dynamics

In regions where the pathogen is endemic, wild animals, particularly rodents and non-human primates, act as the natural reservoirs for the pathogen. Humans become infected via various means, including hunting, handling carcasses, cage cleaning, or the consumption of bushmeat [143]. The 2022 outbreak, however, provided the initial evidence of anthropozoonotic transmission (human-to-animal spillback). A notable case involved an Italian greyhound living with two individuals diagnosed with monkeypox. The dog developed cutaneous and mucosal lesions twelve days after the onset of symptoms in its owners. Genetic sequencing revealed a 100% sequence identity between the human and canine viral isolates, thereby confirming definitive reverse zoonosis [144].

In the Democratic Republic of the Congo (DRC), clinical cohorts have documented cases of spontaneous abortions and fetal deaths in pregnant women infected with MPXV. In one particular instance, a stillborn fetus exhibited diffuse cutaneous lesions, thereby substantiating the occurrence of transplacental viral transmission. Perinatal transmission during delivery or close postpartum contact has also been confirmed, as evidenced by the case observed in the United Kingdom [145].

To date, there is no definitive epidemiological evidence supporting fecal–oral transmission. After a careful examination of all the variables considered, it becomes evident that MPXV has undergone a series of adaptations through time. These adaptations can be attributed to two main factors: accelerated genetic evolution and shifts in human behavioral networks. The net result of these changes is the development of highly effective transmission mechanisms, which have enabled MPXV to spread rapidly and efficiently [146].

### 3.8. Risk Assessment of Hantavirus Spillover

#### 3.8.1. Morphology and Genomic Architecture

Hantaviruses, classified within the family *Hantaviridae* and the genus *Orthohantavirus*, are enveloped viruses characterized by a spherical shape with a diameter ranging from 80 to 120 nanometers. The viral genome consists of three negative-sense, single-stranded RNA segments, designated as S (small), M (medium), and L (large). These segments encode the nucleoprotein (N), the envelope glycoproteins (Gn and Gc), and the viral RNA-dependent RNA polymerase (L protein), respectively. Hantaviruses are susceptible to adverse physicochemical conditions, such as heat, UV radiation, detergents, and organic solvents, which induce viral inactivation. Rodents serve as the primary natural reservoirs for hantaviruses, exhibiting both persistent and largely asymptomatic infections [147]. The initial documentation of hantaviruses was recorded in 1978, during investigations into Korean hemorrhagic fever, a pathology predominantly afflicting rural populations during and following the Korean War. The definitive evidence was obtained via detection of viral antigens in the lung tissues of *Apodemus agrarius*, a rodent species endemic to the affected area, thereby establishing its role as a natural reservoir [147]. Hantaviruses are distinguished by their strict host specificity, which refers to their capacity to preserve an evolutionary association with particular species of reservoir rodents over extended periods. This phenomenon is indicative of a long-term process of virus–host coadaptation, wherein the virus and its host have adapted to each other over successive generations [148]. It is important to note that notable examples of such viruses include the Hantaan virus, which is associated with *Apodemus agrarius*, and the Sin Nombre virus, which is associated with *Peromyscus maniculatus*. Although occasional spillover events into non-reservoir species have been documented, such infections do not generally result in the establishment of sustained transmission cycles [149]. Instead, they are the primary pathway for zoonotic infection in humans.

#### 3.8.2. Zoonotic Spillover and Disease

It is imperative to note that the dynamics of hantavirus spillover are governed by two primary factors: host phylogenetic distance and ecological sympatry. The probability of a successful spillover event increases when non-reservoir species share close evolutionary pathways and overlapping geographic niches with the natural reservoir [150]. From an epidemiological and clinical perspective, hantavirus infections are classified into two primary syndromes: hemorrhagic fever with renal syndrome (HFRS), prevalent in the Old World (Europe, Asia, and Africa), and hantavirus pulmonary syndrome (HPS), characteristic of the New World (the Americas) [151]. In contrast, the Seoul virus (SEOV) exhibits a cosmopolitan distribution. In the Americas, the primary etiological agents implicated in HPS are the Sin Nombre virus (SNV) and the Andes virus (ANDV) [152]. In recent years, mounting epidemiological evidence suggests that the true public health burden of hantavirus infections is significantly underestimated. Recent studies have indicated the presence of underdiagnosis and underreporting in several geographical regions, including Africa, India, and Southeast Asia, where the Thailand virus (THAIV) has also been isolated [153].

#### 3.8.3. Drivers of Zoonotic Spillover Risks

The dynamics of rodent populations are influenced by both climatic and ecological factors, which in turn impact viral transmission. A hypothesis has been postulated that posits a cascade of events, commencing with fluctuations in primary productivity and food resource availability, which modulate host population density, viral circulation, and subsequent human infection risk [154].

A notable example of this phenomenon is the 1993 HPS outbreak in the southern United States. Following the El Niño-Southern Oscillation (ENSO) event, increased rainfall led to an abundance of food resources, causing a population explosion of *Peromyscus maniculatus*, the primary reservoir for the Sin Nombre virus (SNV). This resulted in elevated viral circulation and an upsurge in HPS cases [155]. However, climatic impacts do not follow a uniform global pattern. In other regions, intense precipitation has been observed to disrupt viral transmission dynamics and reduce disease incidence. For instance, in southern China, a negative correlation has been observed between rainfall and rodent population density, resulting in a concurrent decline in the prevalence of HFRS [156]. Human activities contribute to the spread of viruses. Agricultural and urban activities alter host population densities and modify contact between humans and rodents, influencing the spread of infections. Those with frequent exposure to rodent habitats due to their jobs or environment are most at risk. Farmers, hunters, and military personnel are particularly vulnerable. The transmission of the virus occurs through aerosols with viral particles from infected rodents or through an infected rodent bite [7].

Urbanization can reduce viral spread by reducing the fitness of rodents and thereby their reproduction and survival. On the other hand, landscape fragmentation can increase the interface for human transmission [157]. Vaccination is a crucial measure to reduce the spread of the virus. Mass vaccination programs in China have been effective in reducing cases of hemorrhagic fever with renal syndrome (HFRS). This outcome highlights the vital role of immunization in preventing epidemic outbreaks [126]. The transmission of the Andes virus (ANDV) from person to person has been documented on a very limited number of occasions in the context of the Andes virus itself. This transmission modality typically occurs within household clusters under conditions of prolonged close contact, as well as within nosocomial (healthcare) settings. A case of this transmission route was seen in a minor outbreak in Chile in spring 2011, when five cases of hantavirus infection were reported in Corral, in the Los Ríos Region [158,159]. A thorough analysis of molecular and serological data has confirmed the possibility of person-to-person transmission of ANDV. The virus is transmitted through the inhalation of contaminated rodent aerosols, through close contact, and through exposure to body secretions and contaminated materials. These findings contribute to the extant body of knowledge concerning the transmission of ANDV and underscore the significance of infection control measures [160].

### 3.9. Spillover and Spillback: Knowledge Gaps in Biological Drivers and Bidirectional Transmission Dynamics

A metanalysis of emerging infectious diseases (EIDs) from 1940 to the present reveals that 60.3% of these infections are zoonotic in origin, with 71.8% of these events originating specifically from wildlife [161]. The majority of these emerging pathogens possess an RNA genome, which is a structural characteristic that inherently predisposes them to high mutation rates. This genomic instability is primarily driven by the absence of proofreading activity in viral RNA-dependent RNA polymerases (RdRps), which leads to an exceptionally elevated error rate during replication and accelerates adaptive evolution to novel host species [161]. Among wildlife reservoirs, bats and rodents have been demonstrated to exhibit remarkable proficiency in attenuating viral pathology. Bats are particularly noteworthy in this regard, given their ability to tolerate remarkably high viral loads without developing overt clinical disease [162]. This remarkable resilience is achieved by finely balancing immune responses, characterized by constitutive activation of certain interferon pathways in combination with a dampened inflammatory response, such as the down-regulation of the STING (stimulator of interferon genes) pathway. Consequently, they function as optimal, asymptomatic vectors for zoonotic emergence, a phenomenon facilitated by their frequent habitat overlap with humans and livestock [163]. A substantial body of research has documented the presence of a wide variety of highly consequential pathogens—including Hendra, Nipah, Ebola, SARS-CoV, MERS-CoV, and SARS-CoV-2—in bat populations [164]. Despite the substantial scientific progress that has been made in recent decades, a critical knowledge gap remains with respect to the molecular, genetic, and immunological mechanisms that govern the crossing of the final species barrier during a viral spillover event [14]. Although there is considerable documentation of macro-environmental risk factors, including but not limited to anthropogenic climate change, deforestation, biodiversity loss, and inadequate sanitary conditions in low- and middle-income countries (LMICs), the precise intra-host and cellular thresholds required for a virus to breach species barriers remain unknown [165]. This critical gap hinders our capacity to proactively predict which specific viruses demonstrate the greatest potential for imminent spillover. To address this limitation, large-scale metagenomic surveillance initiatives, such as the PREDICT project, have been implemented across 35 countries over an eight-year period. These initiatives have identified more than 1000 novel viruses capable of infecting humans [166,167].

**Figure 4:**
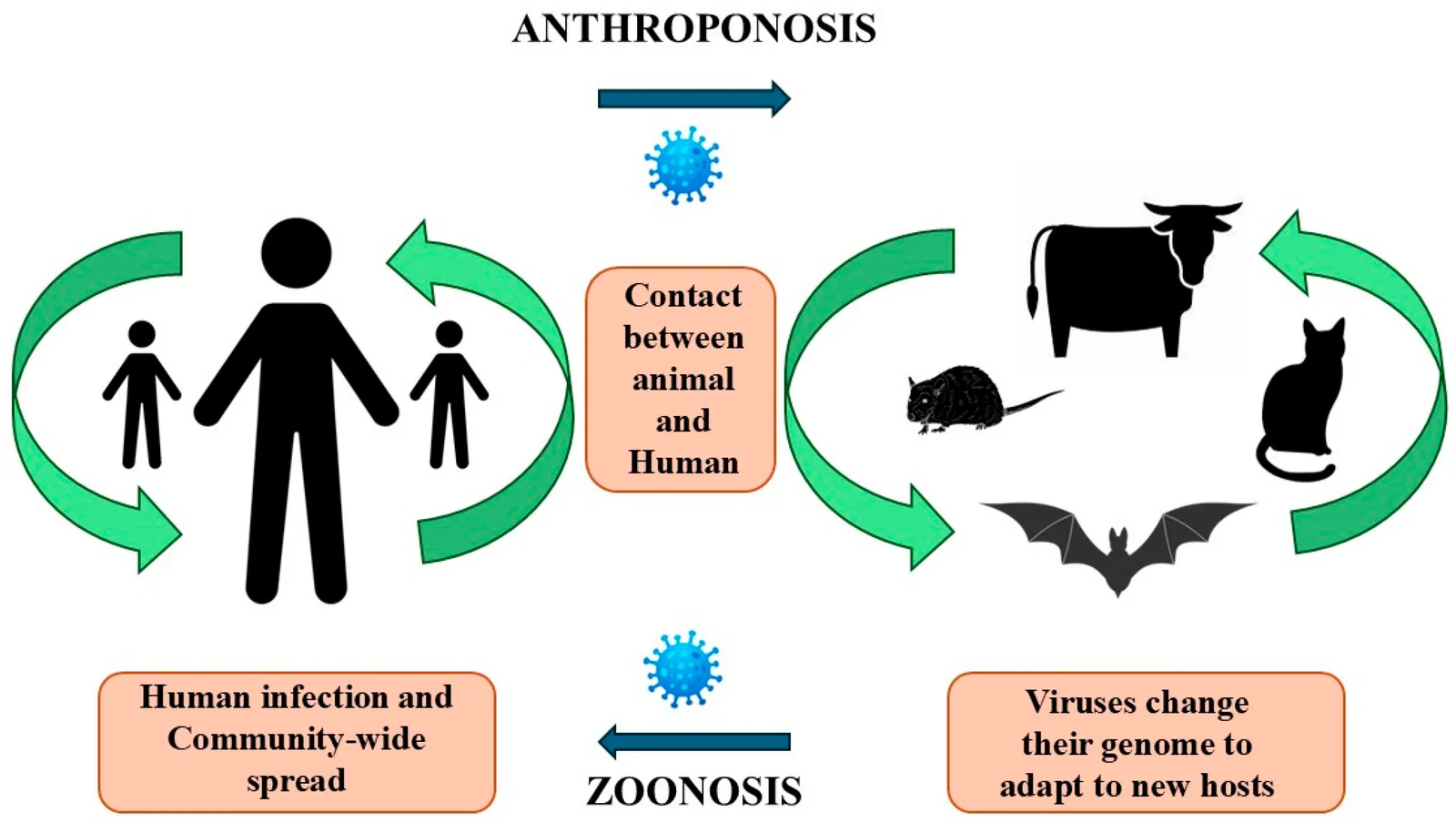
The dynamic and bidirectional nature of viral spillover and spillback. This schematic illustrates the bidirectional relationship between spillover and spillback as a single, dynamic process, moving beyond the traditional paradigm of spillover as a unidirectional event. The critical driver of this mechanism is the close contact between humans and animals, which allows viruses to mutate and adapt their genomes to novel hosts. Once introduced into the human population, the infection disseminates, leading to widespread community transmission. Consequently, viruses exhibit the capacity to jump from animals to humans (spillover/zoonosis) and vice versa (spillback/anthroponosis).

Of particular significance is the contemporary epidemiological paradigm’s transition from conventional, unidirectional frameworks. Contrary to the historical paradigm, in which spillover was considered a one-directional process, recent findings have revealed compelling evidence supporting the existence of a bidirectional dynamic involving reverse zoonosis, also referred to as “spillback.” The bidirectional transmission of this pathogen is exemplified by the case of SARS-CoV-2 [168]. Following its initial emergence among bats and subsequent transmission to humans in Wuhan, China, in late 2019, the virus propagated among farmed and wild mink (Neovison vison) populations in Lithuania by late 2020. The dynamic nature of the interface was confirmed in October 2021, during the global predominance of the Delta variant. Routine human surveillance detected a farm worker infected with the B.1.343 lineage, a strain that had not circulated in the local human population since December 2020. This finding suggests the potential for prolonged, cryptic circulation within the mink population before a subsequent spillover to humans, highlighting the necessity for ongoing epidemiological monitoring to understand the full scope of viral transmission dynamics. An additional well-established example is the phenomenon of anthropogenic spillback of human influenza A viruses into swine populations. Swine respiratory epithelia have been shown to express both avian-like (α-2,3) and human-like (α-2,6) sialic acid receptors. This finding suggests that these animals may serve as a crucial reservoir for viral reassortment (Figure 4).

Genomic surveillance estimates indicate that human seasonal influenza strains are frequently transmitted to pigs at a rate that is drastically underestimated. This bidirectional evolutionary synergy has been identified as a historical driver of the emergence of the 2009 pandemic H1N1 (pdm09) virus, which combined gene segments of human, avian, and swine lineages. This phenomenon persists, manifesting in the emergence of novel swine influenza variants (“v” strains), which pose a persistent zoonotic threat to global public health. This bidirectional evolutionary cycle poses a dual threat: it accelerates biodiversity loss in wildlife and threatens public health by establishing unmonitored animal reservoirs. These reservoirs facilitate the continuous mutation of the virus, which has the potential to compromise human population immunity and the efficacy of vaccines [169,170]. To address these critical gaps, future research must shift beyond static surveillance models. In order to adequately address the issue of spillover and spillback, it is imperative to adopt an integrated framework that conceptualizes these phenomena not as isolated events, but as a continuous, interconnected cycle that operates within the “One Health” paradigm.

## 4. Discussion and Conclusions

The majority of documented cases of viral spillover in mammals are associated with zoonoses, primarily originating from bats, rodents and primates. Three recent epidemics have been caused by animal-borne coronaviruses: SARS, MERS and SARS-CoV-2. However, zoonotic transmission is not exclusive to coronaviruses, since 89% of RNA viruses that cause human illness are zoonotic, with 70% of these viruses originating in wild animals. In recent years, the steadily increasing risk of the spread of zoonotic viruses has become a major cause for concern, primarily due to the impact of human societies on the environment. The repercussions of these actions possess the potential to disturb the delicate equilibrium that exists between humans, animals and their natural environment, thereby augmenting the probability of zoonotic disease transmission. Despite the noteworthy advancements witnessed in recent years in the realm of zoonotic disease research, particularly with respect to the acceleration of early detection and diagnosis (i.e., the expeditious identification of zoonotic viruses such as Ebola and Zika) and the formulation of vaccines and therapeutic interventions for zoonotic diseases (e.g., the development of an efficacious Ebola vaccine and the recent identification of a novel antiviral agent with the potential to combat multiple zoonotic viruses, including Ebola, Lassa fever, and SARS-CoV-2), the necessity to enhance global surveillance and reporting systems to facilitate the expeditious identification and response to outbreaks remains paramount. The increasing incidence of diseases spreading across continents demonstrates the necessity to address health from a global perspective, with efforts to combat these diseases being made in all fields. As humans alter environments, pathogens can spread more easily between animals and people. This is due to the disruption of ecological barriers and deforestation, creating conditions for animal outbreaks and human exposure to zoonoses. Conversion of habitats into farmland brings humans and livestock into contact with wildlife, and urbanization allows prolonged contact with wildlife, facilitating virus transmission. Ecotone environments, shaped by poverty and rapid changes in land use, are the main interface for emerging infectious diseases. Contact between humans and wildlife or livestock creates pathways for zoonotic viruses to adapt to human hosts. Surveillance in regions with many human–animal interactions has identified several worrying viral families, including bat-borne coronaviruses and bird flu. Analyses show that certain viruses already have some compatibility with human physiology and could threaten humans. This underlines the importance of worldwide action to stop the next pandemic. Climate change will make all of this worse by bringing higher temperatures and changes to rainfall. This could mean disease spreading to new areas, carried by mosquitoes or ticks, for example. At the same time, rising temperatures, drought and floods will put pressure on wildlife and public health, making disease more likely. This effect is made worse by more people moving to cities, especially in poor parts of the world. Finally, wildlife trade and live animal markets are high-risk interfaces for zoonotic transmission. Animals involved in these activities are a significant concern as potential reservoirs for novel viruses, especially under conditions of poor sanitation and inadequate disease control. It has been documented that substandard living conditions for animals can contribute to the propagation of infections. Despite the existence of an international trade agreement, few countries currently carry out deep inspections on exported or imported species. On a global scale, there is a paucity of laboratories specializing in the study of animal diseases and the monitoring of epidemics. The centralisation of data could facilitate the comparison of pathogens by species and geographic area, the issuance of alerts regarding mutations within or between hosts, and the identification of differences among viruses. Indeed, ascertaining the viral source is of pivotal importance. It is imperative that efforts are made to train qualified personnel, even in remote areas, who are capable of sequencing viral genomes in specialized laboratories. This approach has already demonstrated its efficacy in dealing with the Ebola and Zika epidemics.

## Figures and Tables

**Table 1:** The epidemiological determinants, reservoirs and case fatality rates of selected emerging viral zoonoses highlight the ecological complexity of spillover events, reinforcing the need for integrated One Health surveillance.

Virus	Initial Outbreak (Year/Origin)	Global Diffusion/Actual	Natural Reservoir	Carrier	CFR (Case Fatality Rate)	References
Ebola	1976, DR Congo/Sudan	West and Central Africa (Global emergency 2026)	Fruit bats (*Pteropodidae*)	None (Direct contact with fluids/bushmeat)	50–90% (Variable depending on the strain)	[20]
West Nile	1937, Uganda (West Nile District)	Africa, Europe, Asia, Americas, Australia	Wild birds (mainly Passeriformes)	Mosquitoes (*Culex* spp.)	<1% (General) ~10% (Neuroinvasive forms)	[21]
SARS-CoV	2002, Guangdong (China)	Contained in 2003 after global spread	Horseshoe bats (*Rhinolophus*)	None (Intermediate host: Civet)	~9–10% (>50% in individuals over 60)	[22]
Zika	1947, Uganda (Zika Forest)	Americas, Africa, Asia, Pacific	Non-human primates/Humans	Mosquitoes (*Aedes* spp.)	<1% (Severe risk of fetal microcephaly)	[23]
H1N1	2009, Messico/USA	Worldwide (Pandemic status 2009)	Swine/Avian	None (Human-to-human airborne transmission)	<0.1%	[24]
Mpox	1970, DR Congo	Central/West Africa, Global (since 2022)	Wild rodents (Hamsters, squirrels)	None (Close contact/lesions)	1–11% (Depending on Clade I or II)	[25]
Yellow fever	Historical, tropical Africa	Tropical regions of Africa and Central-South America	Non-human primates/Humans	Mosquitoes (*Aedes* and *Haemagogus*)	20–50% (In patients with toxic phase)	[26]
MERS-CoV	2012, Saudi Arabia	Arabian Peninsula and imported outbreaks (27 countries)	Dromedaries (*Camelus dromedarius*)	None (Direct contact/Aerosol)	34.4% (866 deaths out of 2519 confirmed cases)	[27]
SARS-CoV-2	2019, Wuhan (China)	Worldwide (COVID-19 pandemic)	Bats (*Rhinolophus*)/Huanan Market	None (Airborne/Aerosol transmission)	~0.5–2% (Variable due to variants and vaccines)	[22]
Chikungunya	1952, Tanzania	Africa, Asia, Americas, Southern Europe	Non-human primates/Humans	Mosquitoes (*Aedes* spp.)	<0.1% (High chronic joint morbidity)	[28]
Crimean-Congo	1944, Crimea	Africa, Balkans, Middle East, Asia	Wild/domestic mammals and birds	Ticks (*Hyalomma* spp.)	10–40%	[29]
Hantavirus	1950–1953 (Korean War)	Eurasia, Americas/Hantavirus Outbreak on a Cruise Ship, 2026	Rodent species (families *Muridae* and *Cricetidae*)	wild rodents, rats, and voles	<1 in Europe and up to 40% in the Americas	[30,31]

## Data Availability

No new data were created or analyzed in this study.
